# Novel randomization and iterative based algorithms for the transactions assignment in blockchain problem

**DOI:** 10.1371/journal.pone.0286667

**Published:** 2023-06-21

**Authors:** Abdullah Bajahzar

**Affiliations:** Department of Computer Science and Information, College of Science at Zulfi, Majmaah University, Al-Majmaah, Saudi Arabia; Universiti Teknologi Malaysia, MALAYSIA

## Abstract

This study focuses on the load balancing of the transactions in the blockchain. The problem is how to assign these transactions to the blocks. The objective is to guarantee a load balancing of the workload in the time of blocks. The proposed problem is an NP-hard one. To face the hardness of the studied problem, the challenge is to develop algorithms that solve the problem approximately. Finding an approximate solution is a real challenge. In this paper, nine algorithms are proposed. These algorithms are based on the dispatching-rules method, randomization approach, clustering algorithms, and iterative method. The proposed algorithms return approximate solutions in a remarkable time. In addition, in this paper, a novel architecture composed of blocks is proposed. This architecture adds the component “Balancer”. This component is responsible to call the best-proposed algorithm and solve the scheduling problem in a polynomial time. In addition, the proposed work helps users to solve the problem of big data concurrency. These algorithms are coded and compared. The performance of these algorithms is tested over three classes of instances. These classes are generated based on uniform distribution. The total number of instances tested is 1350. The average gap, execution time, and the percentage of the best-reached value are used as metrics to measure the performance of the proposed algorithms. Experimental results show the performance of these algorithms and a comparison between them is discussed. The experimental results show that the best algorithm is best-mi-transactions iterative multi-choice with 93.9% in an average running time of 0.003 s.

## 1 Introduction

Nowadays, the utilization of blockchain technology is in increase day after day [1]. Person use of blockchain technology has also remarkably increased since 2016. The statistics published by several sources in 2020 show that there were more than 40 million blockchain wallets this year in comparison to 10 million blockchain wallets in 2016. The blockchain is integrated into several areas. Indeed, cryptocurrencies are used in a large way the blockchain technology. The security of cryptocurrencies in blockchain technology is studied giving state-of-art challenges and future prospects in [2]. Various category is presented in the latter work to show the importance of the blockchain applied to cryptocurrencies.

In the face of multiple transactions and a huge number of transactions, good management of these transactions optimize the use of the blocks to reply rapidly to all requested demands. Each transaction is characterized by its estimated execution time. In this paper, we focus on the load balancing of the transactions to the different blocks. The problem addressed in this paper is novel because the work reported has not been proposed or studied anywhere else in the literature. A solution is proposed for a new industrial problem that can be used to balance blocks into blockchain technology.

This paper proposes nine algorithms to find the best way to assign the big number of transactions in the given blocks, ensuring fair time execution between blocks. These algorithms are based on the dispatching-rules method, randomization approach, clustering algorithms, and iterative method. The proposed algorithms return approximate solutions in a remarkable time.

The choice to use heuristics rather than other methodologies is based on the following advantages provided by heuristic-based algorithms:

1. Simplicity: Heuristics are usually simpler and easier to interpret and understand. Heuristic algorithms often involve many sets of logical rules that the researcher can manipulate easily.
2. Efficacy: Heuristic algorithms can be faster (time execution) and need less memory to expand than other models.
3. Robustness: Heuristics are also more robust than other models.
4. Area-particular knowledge: Heuristics may encompass area-particular knowledge and proficiency, which can be difficult to capture in an other model.

A comparative study was discussed to measure and show the effectiveness of each developed algorithm compared to the best solutions among all proposed algorithms.

The essential contributions of this work are detailed in the following points:

- The proposal and coding of nine algorithms to solve an NP-hard problem.
- The design of a novel architecture that adds a new component called “Balancer”.
- The running time to obtain a solution to the studied problem is polynomial.
- There is no dominance between the algorithms. This means that any combination of two or more algorithms gives a new result. This can give authors more flexibility to choose several combinations and amelioration to extend the work and utilize the proposed algorithms.
- The proposed algorithms show their performance over different classes of instances.
- The utilization of the proposed algorithms as initial solutions to apply different meta-heuristics to solve the studied problem.

The rest of the paper is organized into six sections. A stat of the art is detailed and analysed in Section 2. The second section is reserved for the architecture composed of blocks. In this section, a novel architecture is given and details the different components. A new component called “Balancer” is proposed. The third section defines the problem formulation. All used variables and the modernization of the objective function are detailed in this section. The fourth section presents approximate solutions through different proposed algorithms. Nine algorithms are proposed and illustrated. Section 6 reports the results and discussions. Three classes of instances are tested to compare the performance of the proposed algorithms. Finally, the paper concludes with a summary in Section 7.

## 2 State of the art

In [3], the authors give different national and international perspectives regarding the use of cryptocurrencies. The discussion of the cryptocurrency innovations such as Bitcoin is detailed in [4]. A discussion of the non-trusted blockchain technology called Bitcoin. These authors presented three characterizations of digital money Bitcoin. In [5] authors give an overview and future perspectives on the utilization of the blockchain in cryptocurrency. In addition, the authors analyze current cryptocurrency projects and give different examples. In this same context authors in [6–9] studied another literature review regarding the blockchain in cryptocurrency. Blockchain technology is applied in different domains. Indeed, several works treated the application of blockchain technology. In [10–12] authors presented a survey of blockchain applications in different domains. Offered contracts that apply or perform fully or partially automatically without human intervention. These contracts are called smart contracts [13]. Several works treated blockchain-based smart contracts. Indeed, in [14], the authors extracted 24 papers from several scientific databases to track the smart contract issues and analyses the impact of this utilization. Different smart contract applications are illustrated and future studies are provided. In [15], the authors analyze 468 research papers that studied the smart contract and their 20,188 references. Six major streams related to the studied research area are identified using factor analysis. Authors in [16] explained the diverse components and working fundamentals of the smart contract. In addition, the authors identified and analyzed the diverse use cases of smart contracts with the interest of utilizing smart contracts in blockchain in several domains. Different algorithms are illustrated related to the use of logic in smart contracts with blockchain systems [17]. In healthcare management, several existing in previous research work and applications studied for the healthcare domain using the blockchain approach [18]. Other surveys regarding the contracts with blockchain are studied by several researchers [19, 20]. In [21], authors explain the combination between the blockchain and the IoT. The authors based the work on the sharing of services and resources and a cryptographically verifiable manner. In fact, this latter work shows that the blockchain-IoT combination can give the new world a new vision regarding real-life manipulation. Another domain that the blockchain technology is applied is financial services. In this context, the blockchain and its effect on financial sectors are treated in [22]. The load balancing is the main core of the studied problem. The load balancing algorithms are largely studied in the literature in different domains in [23–27].

A comprehensive study of the previous research work regarding this issue is presented in [28]. Several works detailed the financial services related with blockchain in [29–32]. The supply chain with the utilization of the blockchain is also studied in the literature. In [33], authors show how the boundary conditions must be met before blockchain can be applied. Eighteen boundary conditions were proposed. The shifting trust in the creation of supply chains via blockchain is studied in [34]. A case study of the data management in supply chains using blockchain is presented in [35]. In our work, several algorithms are proposed to solve the load balancing in the blockchain. On the other hand, several works in literature treated the load balancing or as also known as the fair distribution or the equity distribution [36–40].

Several papers treated the blockchain used different methods that can exploit the proposed algorithms to extend their works [41–46].

The proposed algorithms can be adopted to solve the parallel machine problem studied in [47–52].

Table 1 provides a summary of the related works detailed previously.

**Table 1:** Summary of previous work.

Ref	Type	Purpose	Area	Methodology
[3]	Book	Cryptocurrencies and Types of Cryptocurrencies	Financial Services Technology	Digital technology
[4]	Research	Cryptocurrency innovations	Non-trusted blockchain technology	Digital technology
[5–9]	Survey	Utilization of the blockchain in cryptocurrency	Financial Services Technology	Future perspectives of cryptocurrencies and blockchain technology
[10]	Survey	Study to motivate more blockchain applications	cryptocurrency, healthcare, advertising, insurance, copyright protection, energy, and societal applications	Security and privancy
[11]	Survey	Identifying the major fields of study and areas of application for blockchain	Internet of Things, energy, finance, healthcare, and government	Blockchain technology, cryptocurrencies, and Bitcoin
[12]	Resaerch	The use of blockchain technologies	Industrial application domains	Blockchain technology
[13]	Book	Understanding Bitcoin	Cryptography, Engineering and Economics	Transaction processing, smart contracts and mining technologies
[14–16]	Survey	Blockchain-based smart contracts	Smart contract technologies	Smart contract
[17]	Research	Use of logic-based smart contracts in combination with blockchain systems	Smart contract technologies	Algorithms from defeasible logic-based frameworks
[18]	Research	Propose multiple workflows involved in the healthcare ecosystem using blockchain	Healthcare Management	Workflows of the medical smart contract system
[19, 20]	Survey	Blockchain smart contracts	Smart contracts	Security verification of blockchain smart contracts
[21]	Research	Combination between the blockchain and the IoT	Blockchain-IoT combination	Cryptographically verifiable manner
[22]	Research	How “Blockchains” work	Blockchain	Blockchain and Bitcoin network
[24, 27, 37, 39]	Research	Projects distribution	Management of projects	Algorithms and heuristics
[26]	Research	Done-battery optimization	Management of drone missions	Algorithms and heuristics for load balancing
[25, 38]	Research	Parking optimization	Management of parking	Algorithms and heuristics for load balancing
[28]	Research	Review studies that have used blockchain technology in financial services	Namely, computational logic, peer-to-peer transmission, irreversibility of records, distributed database and transparency	Use of blockchain technology in financial services
[29]	Research	Blockchain technology	blockchain technology	Security
[30]	Research	Governance practices of established or popular blockchain and decentralized autonomous organization	Financial services	Key governance issues in financial infrastructure
[31]	Research	Blockchain technology	Global Financial Services Industry	Digital technology
[32]	Research	Contemporary BlockChain platforms in financial services	BlockChain platforms	Computer technology without human intervention
[32]	Research	Blockchain can result in more information sharing	Blockchain technology	Boundary conditions
[34]	Research	Supply chains via blockchain	Blockchain technology	Shifting trust in the creation of supply chains
[35]	Research	Supply chains and blockchain	Blockchain technology	Data management in supply chains using blockchain
[36]	Research	Doctors workload	Healthcare management system	Load balancing algorithms
[40]	Research	Manufacturing machine management	Industry	Load balancing algorithms

Several other works treating the blockchain problem can be considered [46, 53, 54].

Other scheduling algorithms treated in [55–57] can be exploited to deal with the studied problem.

These previous works give many limitations that can be detailed as follows:

- Scalability: some algorithms can not be easily scalable;
- Overhead: Several presented algorithms related to load balancing can give additional overhead;
- insufficiency of accuracy: some Load-balancing algorithms may not always accurately forecast the load on blocks;
- Limitation of implementation: Some algorithms may only be applicable to certain types of transactions, servers, and models.

To surmount these limitations, this paper proposes nine novel algorithms for efficacy manage the very high number of transactions to the different offered blocks. These algorithms are based on the dispatching-rules method, randomization approach, clustering algorithms, and iterative method. The proposed algorithms return approximate solutions in a remarkable time.

## 3 Architecture composed of blocks

**Fig 1:**
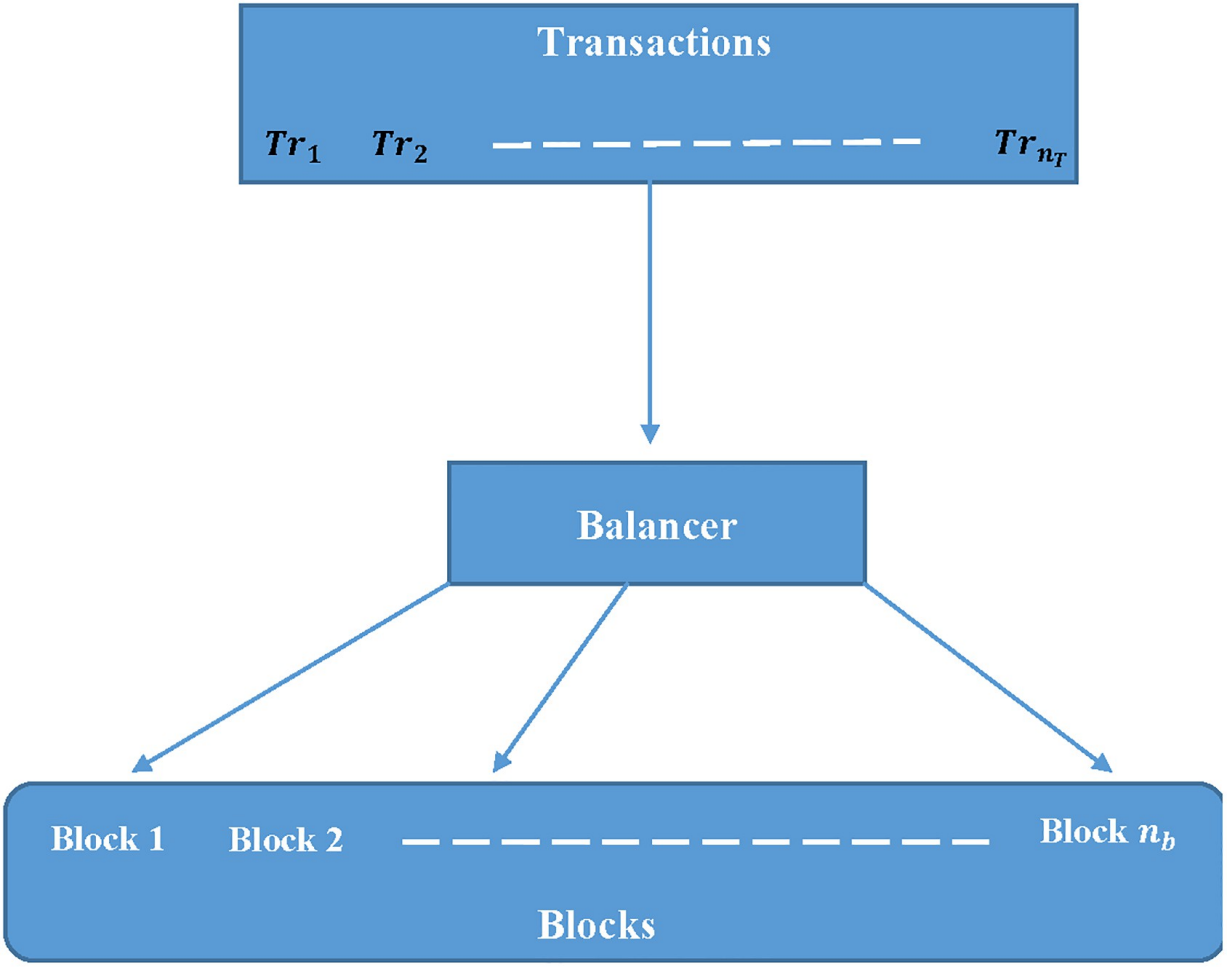
Architecture of the blocks-transactions assignment problem.

The studied problem is based on the assignment of the different transactions to different blocks. Fig 1 illustrates an overview of this problem. As shown in this latter figure, the component “Balancer” is a novel component that is added to the proposed architecture. This component is responsible to run the proposed algorithms and choose the best one to decide how to assign the given transactions to different blocks. The “Balancer” solve an NP-hard problem. For this reason, the execution time of the algorithm is very crucial and must be considered as one of the metrics to choose the effeminacy of the algorithm.

The proposed architecture is composed of three components detailed as follows:

- Transactions
- Balancer
- Blocks

The transactions component is the engine that is responsible to collect all the given transactions that must be executed. In addition, this component collects also all information on each transaction essentially the estimated running time of each transaction. The second component is the “Balancer”. This is the core of the architecture. Several algorithms are called to solve the problem constituted by the given transactions. Finally, the component “Blocks” is composed of the available blocks.

## 4 Problem formulation

Let *Tr* be a set of *n*_*T*_ transactions to be assigned to *n*_*b*_ blocks into a blockchain. Each transaction *j* satisfies certain characteristics. The estimated running time *rt*_*j*_ for each transaction is fixed in advance. The cumulative estimated running time when the transaction *j* is assigned is *Cr*_*j*_ ∀*j* ∈ {1, …, *n*_*T*_}. The total workload for each block *i* after finishing the assignment is *Tw*_*i*_ ∀*i* ∈ {1, …, *n*_*b*_}. The maximum completion time when all transactions are completed is *Tw*_*max*_ and formulated in Eq 1.

$$\begin{array}{c} Tw_{max}=\underset{1\leq j\leq n_{T}}{\text{max}}\;Cr_{j} \end{array}$$ (1)

The maximum completion time *Tw*_*max*_ can be formulated as described in Eq 9.

$$\begin{array}{c} Tw_{max}=\underset{1\leq i\leq n_{b}}{\text{max}}\;Tw_{i} \end{array}$$ (2)

The total completion time gap is denoted by *Gtw* and shown in Eq 3.

$$\begin{array}{c} Gtw=\sum_{k=1}^{n_{b}}\left[Tw_{max}-Tw_{k}\right] \end{array}$$ (3)

The goal is to find a solution that minimizes the sum of the gaps between the block that has the minimum execution time and each other blocks. The objective is to minimize *Gtw*.

We define the variable *x*_*ij*_ as detailed in Eq 4.

$$\begin{array}{c} x_{ij}=\{\begin{array}{cc} 1 & \text{if}\;\text{transaction}\;j\;\text{is}\;\text{scheduled}\;\text{to}\;\text{block}\;i,\\ 0 & \text{Otherwise}. \end{array} \end{array}$$ (4)

The constraints of the proposed problem are detailed in Eqs 5, 6, 7 and 8.

$$\{\begin{array}{lll} \sum_{i=1}^{n_{b}}x_{ij}=1,\forall j\in \{1,\cdots ,n_{T}\} & & \left(5\right)\\ \sum_{j=1}^{n_{b}}rt_{j}x_{ij}\leq Tw_{max},\forall i\in \{1,\cdots ,n_{b}\} & & \left(6\right)\\ x_{ij}\in \{0,1\},\forall i\in \{1,\cdots ,n_{b}\}\;\text{and}\;\forall j\in \{1,\cdots ,n_{T}\} & & \left(7\right)\\ Tw_{max}>0 & & \left(8\right) \end{array}$$

**Example 1** *Assume that the number of transactions is 7 and the number of blocks is 2. This example considers only the transactions to be scheduled during a period of time. For other time periods, a new set of transactions will be scheduled on the two blocks*. Table 2 *lists the estimated running time for each transaction*.

**Table 2:** 7-transactions and 2-blocks instances.

*j*	1	2	3	4	5	6	7
*rt*_*j*_	5	3	10	2	9	6	4

**Fig 2:**
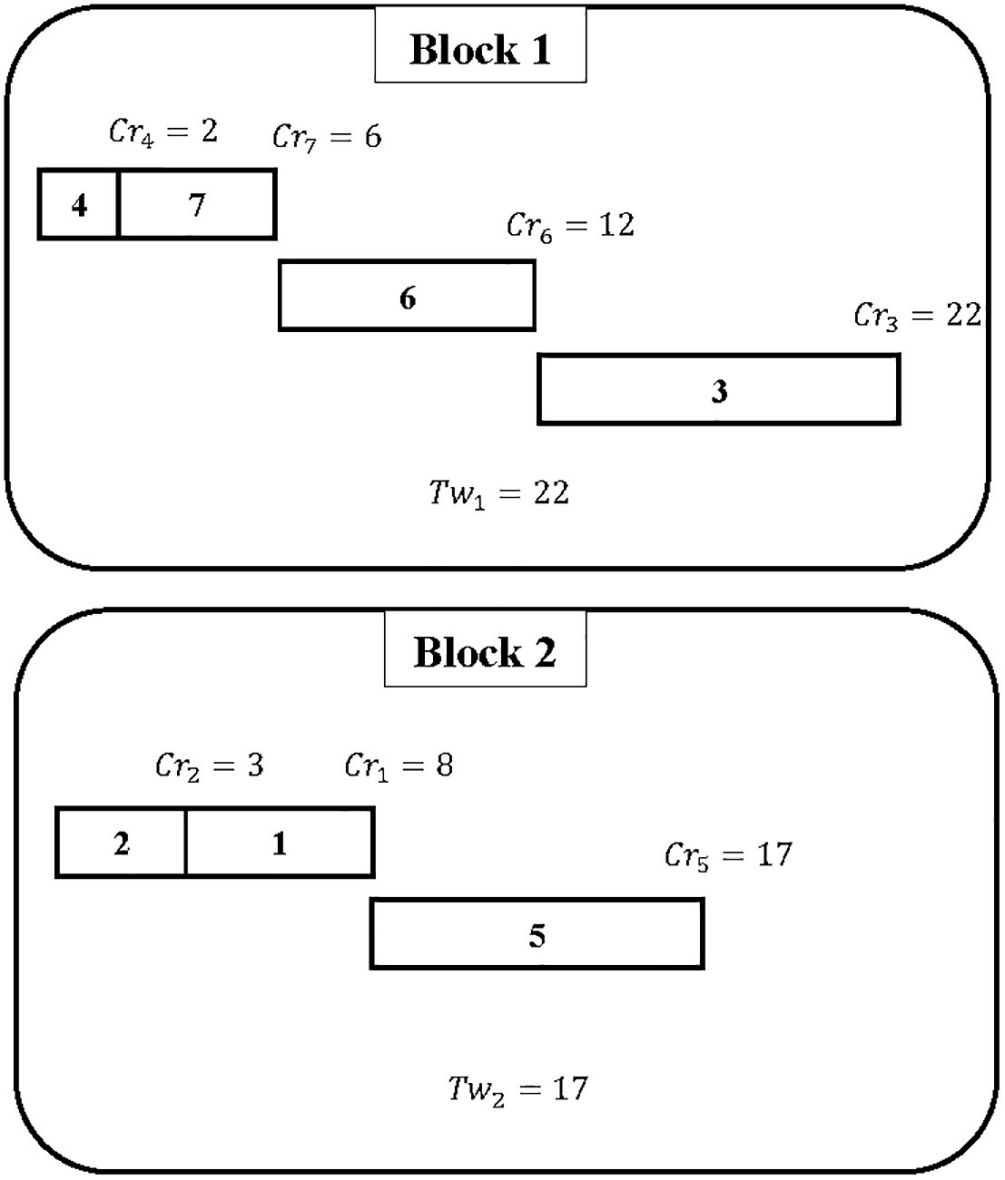
Schedule for Example 1.

*The objective is to find a solution that will assign all the transactions on the two given blocks*. Fig 2 *illustrates an example of a schedule regarding the feasible solution to the studied problem*.

*The schedule given in* Fig 2 *is as follows: On block 1, transactions* {4, 7, 6, 3} *are executed, while on block 2, transactions* {2, 1, 5} *are executed*. *From* Fig 2, *it can be deduced that the first block has a total execution time of 22 and the second block has a total execution time of 17. The gap between the total execution times for block 1 and block 2 is equal to Tw*_1_ − *Tw*_2_ = 5. *The principal goal of the work reported here is to minimize this gap. Therefore, a more efficient assignment needs to be found with a gap of less than 5. To calculate the gap between the blocks, several indicators are chosen. In this paper and for the above example the following indicator is proposed: Tw*_1_ − *Tw*_2_ *where Tw*_1_ *represents Tw*_*max*_.

**Fig 3:**
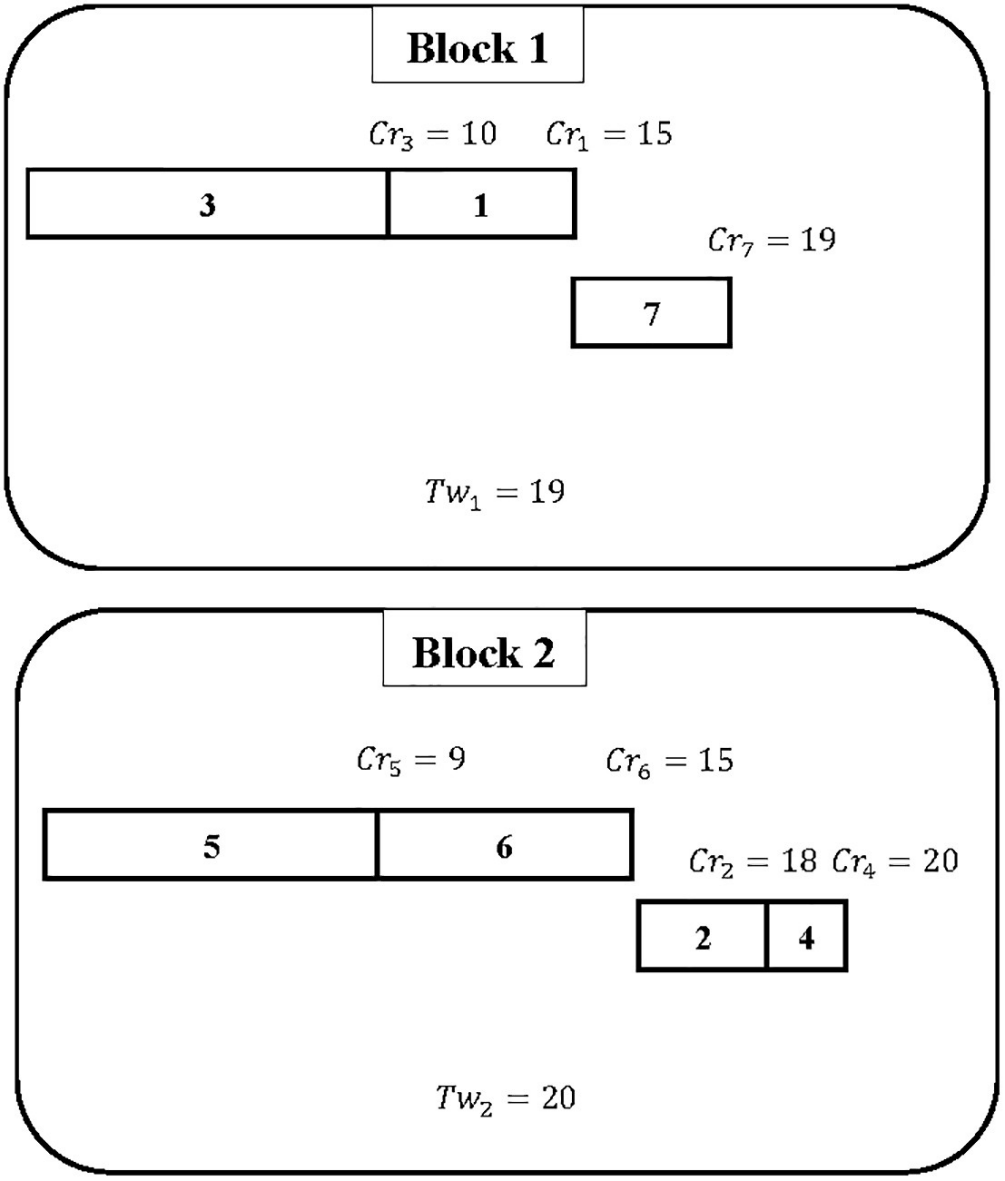
Schedule for Example 2.

**Example 2** *Assume that we have the same instance detailed in* Table 2. Fig 3 *illustrates another example of a schedule regarding the feasible solution of the studied problem*.

*The schedule given in* Fig 3 *is as follows: On block 1, transactions* {3, 1, 7} *are executed, while on block 2, transactions* {5, 6, 2, 4} *are executed. From* Fig 3, *it can be deduced that the first block has a total execution time of 19 and the second block has a total execution time of 20. The gap between the total execution times for block 1 and block 2 is equal to Tw*_2_ − *Tw*_1_ = 1. *The principal goal of the work reported here is to minimize this gap. This schedule shows that a more efficient assignment is found with a gap of less than 5 compared with the schedule in Example 1*.

## 5 Proposed algorithms

In this section, we detail all the developed algorithms regarding the studied problem of the blockchain with constraints. Nine algorithms are proposed. The first algorithm is called “Longest Transactions Time” and is denoted by *LTT*. This algorithm is based on the dispatching rule. The second algorithm is called “Smallest Transactions Time” and is denoted by *STT*. This algorithm is based on the dispatching rule. A probabilistic algorithm called “Iterative Multi-choice Longest-Transactions Time” and denoted by *IML* is presented as the third algorithm. The fourth algorithm is called “Iterative Multi-choice Smallest-Transactions Time” and is denoted by *IMS*. This algorithm is based on the iterative method. The fifth algorithm is called “Block-transaction Iterative Longest-Multi-choice” and is denoted by *BIL*. This algorithm is based on the probabilistic approach. The “Block-transaction Iterative Smallest-Multi-choice” algorithm is denoted by *BIS* and constitutes the sixth algorithm. The seventh algorithm is called “Mi-transactions Iterative Longest-Multi-choice” and is denoted by *MIL*. The “Mi-transactions Iterative Smallest-Multi-choice” algorithm is denoted by *MIS* and is the eighth one. The last algorithm is called “Best-mi-transactions Iterative Multi-choice” and is denoted by *BIM*. All algorithms receive as inputs the number of blocks *n*_*b*_, the number of transactions *n*_*T*_, and the values of the estimated running time *rt*_*j*_ for each transaction. The proposed algorithms are independent and different from each other.

### 5.1 Longest Transactions Time (*LTT*)

This algorithm uses the dispatching rule method by sorting all transactions according to the non-increasing order of their estimated running time. After the sorting, the scheduling will be on the block that has the minimum total workload. The sorting algorithm used for the dispatching rule is the heapsort algorithm. The complexity of this algorithm is *O*(*nlogn*) [57].

### 5.2 Smallest Transactions Time (*STT*)

This algorithm is the same as *LTT*, the difference is to sort all transactions according to the non-increasing order of their estimated running time.

### 5.3 Iterative Multi-choice Longest-Transactions Time (*IML*)

The transaction that has the first longest running time and the transaction that has the second longest running time. This is meaning that the choice will be between the two first elements in the table of transactions after sorting. This choice will be based on the application of the probability *μ*. In fact, for *μ* probability, the choice is for the transaction that has the first longest running time and for 1 − *μ* probability for the transaction that has the second longest running time. In the practice, *μ* = 0.4. The choice will be repeated *itn* = 800 times and the best solution will be chosen. The procedure *NIO*() is responsible for the ordering of the transactions given in input according to the non-increasing order of its running time. The procedure *SHE*() is responsible for the scheduling of the transactions given in input on the most available block. In addition, *Tr*_1_ represents the first transaction that has the longest running time and *Tr*_2_ represents the second transaction that has the longest running time. Algorithm 1 represents the algorithm of *IML*.

**Algorithm 1** Iterative Multi-choice Longest-Transactions Time Algorithm (*IML*)

1: Call *NIO*(*Tr*)

2: **for** (*h* = 1 to *itn*) **do**

3:  **for** (*k* = 1 to *n*_*T*_ − 1) **do**

4:   Determine *Tr*_1_ and *Tr*_2_

5:   **if** (*μ*) **then**

6:    Call *SHE*(*Tr*_1_)

7:   **else**

8:    **if** (*k* = *n*_*T*_ − 1) **then**

9:     **if** (*Tr*_1_ is not scheduled) **then**

10:      Call *SHE*(*Tr*_1_)

11:     **else**

12:      Call *SHE*(*Tr*_2_)

13:     **end if**

14:    **end if**

15:   **end if**

16:  **end for**

17:  Calculate *Gtw*_*h*_

18: **end for**

19: Calculate $Gtw=\underset{1\leq h\leq itn}{\text{min}}\;Gtw_{h}$

20: Return *Gtw*

### 5.4 Iterative Multi-choice Smallest-Transactions Time (*IMS*)

This algorithm is similar to *IML*. The difference is to sort the transactions in the first step according to the non-decreasing order of its *rt*_*j*_.

### 5.5 Block-transaction Iterative Longest-Multi-choice (*BIL*)

This algorithm uses the randomization method. The first step is to sort all transactions according to the non-increasing order of its *rt*_*j*_. Next, the second step is to schedule the *n*_*b*_ transactions that have the longest running time to the blocks. The third step is concerning the remaining transactions. The remaining transactions will be scheduled with a probability *ϑ*. This probability is based on the choice of the transaction. In fact, for *ϑ* probability, the choice is for the transaction that has the first longest running time and for 1 − *ϑ* probability for the transaction that has the second longest running time. In practice, *ϑ* = 0.4. The choice will be repeated *itn* = 800 times and the best solution will be picked. The set *LT* contains the first *n*_*b*_ transactions that have the longest running time. Algorithm 2 represents the algorithm of *BIL*.

**Algorithm 2** Block-transaction Iterative Longest-Multi-choice Algorithm (*BIL*)

1: Call *NIO*(*Tr*)

2: **for** (*h* = 1 to *itn*) **do**

3:  **for** (*k* = 1 to *n*_*T*_ − *n*_*b*_) **do**

4:   Determine *Tr*_1_ and *Tr*_2_

5:   **if** (*μ*) **then**

6:    Call *SHE*(*Tr*_1_)

7:   **else**

8:    Call *SHE*(*Tr*_2_)

9:   **end if**

10:   **if** (*k* = *n*_*T*_ − *n*_*b*_) **then**

11:    **if** (*Tr*_1_ is not scheduled) **then**

12:     Call *SHE*(*Tr*_1_)

13:    **else**

14:     Call *SHE*(*Tr*_2_)

15:    **end if**

16:   **end if**

17:  **end for**

18:  Calculate *Gtw*_*h*_

19: **end for**

20: Calculate $Gtw=\underset{1\leq h\leq itn}{\text{min}}\;Gtw_{h}$

21: Return *Gtw*

### 5.6 Block-transaction Iterative Smallest-Multi-choice (*BIS*)

This algorithm uses the randomization method. The first step is to sort all transactions according to the non-increasing order of its *rt*_*j*_. Next, the second step is to schedule the *n*_*b*_ transactions that have the longest running time to the blocks. The third step is concerning the remaining transactions. The remaining transactions will be scheduled with a probability *ϑ*. This probability is based on the choice of the transaction. In fact, for *ϑ* probability, the choice is for the transaction that has the first smallest running time and for 1 − *ϑ* probability for the transaction that has the second smallest running time. In practice, *ϑ* = 0.4. The choice will be repeated *itn* = 800 times and the best solution will be picked. We denoted by *TrS*_1_ represents the first transaction that has the smallest *rt*_*j*_ and *TrS*_2_ represents the second transaction that has the smallest *rt*_*j*_. Algorithm 3 represents the algorithm of *BIS*.

**Algorithm 3** Block-transaction Iterative Smallest-Multi-choice Algorithm (*BIS*)

1: Call *NIO*(*Tr*)

2: Call *SHE*(*LT*)

3: **for** (*h* = 1 to *itn*) **do**

4:  **for** (*k* = 1 to *n*_*T*_ − *n*_*b*_) **do**

5:   Determine *TrS*_1_ and *TrS*_2_

6:   **if** (*μ*) **then**

7:    Call *SHE*(*TrS*_1_)

8:   **else**

9:    Call *SHE*(*TrS*_2_)

10:   **end if**

11:   **if** (*k* = *n*_*T*_ − *n*_*b*_) **then**

12:    **if** (*TrS*_1_ is not scheduled) **then**

13:     Call *SHE*(*TrS*_1_)

14:    **else**

15:     Call *SHE*(*TrS*_2_)

16:    **end if**

17:   **end if**

18:  **end for**

19:  Calculate *Gtw*_*h*_

20: **end for**

21: Calculate $Gtw=\underset{1\leq h\leq itn}{\text{min}}\;Gtw_{h}$

22: Return *Gtw*

### 5.7 Mi-transactions Iterative Longest-Multi-choice (*MIL*)

The first step is to sort all transactions in the non-increasing order of its *rt*_*j*_. After that, the second step is to schedule the $\frac{n_{T}}{2}$ transactions that have the longest running time to the blocks. The third step is concerning the remaining transactions. The remaining transactions will be scheduled with a probability *ϑ*. This probability is based on the choice of the transaction. In fact, for *ϑ* probability, the choice is for the transaction that has the first longest running time and for 1 − *ϑ* probability for the transaction that has the second longest running time. In practice, *ϑ* = 0.4. The choice will be repeated *itn* = 800 times and the best solution will be picked. We denoted by *SHEST*(*L*, *TwI*) is the procedure that schedules the transactions in the list *L* taking into consideration the load of blocks *Tw*_*i*_ stored into the list *TwI*. This means that the blocks are not initially empty. The instructions of *MIL* are detailed in Algorithm 4.

**Algorithm 4** Mi-transactions Iterative Longest-Multi-choice algorithm (*MIL*)

1: Call *NIO*(*Tr*)

2: **for** (*j* = 1 to $\frac{n_{T}}{2}$) **do**

3:  Call *SHE*(*Tr*_*j*_)

4: **end for**

5: Determine *TwI*

6: Set *Twa* = *TwI*

7: **for** (*k* = 1 to *itn*) **do**

8:  **for** ($h=\frac{n_{T}}{2}+1$ to *n*_*T*_) **do**

9:   Select the transaction *Tr* based on *ϑ*

10:   Call *SHEST*(*Tr*, *TwI*)

11:  **end for**

12:  Calculate *Gtw*_*k*_

13:  Rest *TwI* = *Twa*

14: **end for**

15: Calculate $Gtw=\underset{1\leq k\leq itn}{\text{min}}\;Gtw_{k}$

16: Return *Gtw*

### 5.8 Mi-transactions Iterative Smallest-Multi-choice (*MIS*)

The same procedure is like the *MIL* algorithm, however, the difference is regarding the remaining transactions after the scheduling of the $\frac{n_{T}}{2}$ transactions that have the longest running time to the blocks. In the third step, instead of the transaction that has the first longest running time for probability *ϑ*, we select the transaction that has the first smallest running time.

### 5.9 Best-mi-transactions Iterative Multi-choice (*BIM*)

Firstly, we call the iterative multi-choice longest-transactions time algorithm and we call the block-transaction iterative longest-multi-choice algorithm. The best solution will be selected. Thus, we have the following equation:

$$\begin{array}{c} BIM=Min(IML,BIL) \end{array}$$ (9)

We denoted by *Gtw*_*I*_ and *Gtw*_*B*_ the total completion time gap returned by *IML* and *BIL*, respectively. Algorithm 5 represents the algorithm of *BIM*.

**Algorithm 5** Best-mi-transactions Iterative Multi-choice algorithm (*BIM*)

1: Call *IML*(*Tr*)

2: Call *IML*(*Tr*)

3: Set *Gwt* = min(*Gtw*_*I*_, *Gtw*_*B*_)

4: Return *Gwt*

## 6 Results and discussion

In this section, we detail the obtained results after running all proposed algorithms. The proposed algorithms were codded using a C++ program using Visual Studio 2019. The hardware used is an Intel(R) Core(TM) i5-6200U CPU @ 2.30GHz 2.40 GHz and 8 GB RAM. The operating system is Windows 10 Enterprise with 64-bit version 22H2.

The experimental results show different advantages of the proposed algorithms as follows:

- The execution time to obtain a solution to the studied problem is polynomial.
- There is no dominance between the algorithms. This means that any combination of two or more algorithms gives a new result. This can give authors more flexibility to choose several combinations and amelioration to extend the work and utilize the proposed algorithms.
- The proposed algorithms show their performance over different classes of instances.
- The utilization of the proposed algorithms as initial solutions to apply different met-heuristics to solve the studied problem.

Despite the advantage of the proposed algorithms, there are some limitations of the research work as follows:

- The work develops novel algorithms and architecture based on the component “Blancer”. However, the work doesn’t give a lower bound of the problem to measure the performance of the results compared to the lower bound. Measuring the distance to the lower bound is important to know the far of the proposed algorithms to the optimal solution.
- Some proposed algorithms give a high average gap. The average gap can be a performance by applying other methods.
- The proposed algorithms are not tested in big-scale instances.
- More other distributions can be tested to generate the instances like normal distribution and binomial distribution.

Several instances were generated to perform the proposed algorithms.

These algorithms are compared between them and measured using different metrics. The instances tested in this section are generated like in [38]. Three classes are selected to test the proposed algorithms. These classes are based on the uniform distribution *UnD*[.]. The *rt*_*j*_ of the transaction *j* was generated as:

- Class CS1: *rt*_*j*_ ∈ *UnD*[5, 15].
- Class CS2: *rt*_*j*_ ∈ *UnN*[10, 25].
- Class CS3: *rt*_*j*_ ∈ *UnN*[10, 50].

The number of transactions is varied as {10,15,20,25,30,35,40,45,50} and the number of blocks is varied as {2,3,4,5,6}. For each number of transactions, each number of blocks, and each class, 10 instances were generated. So, in total, we have 1350 instances. Three metrics are proposed to show the performance of the given algorithms. These metrics are:

-
$G=\frac{Gtw-{Gtw}_{b}}{Gtw}$ : the gap obtained based on the best value picked after execution of all algorithms (*Gtw*_*b*_) and the value obtained by the presented algorithm (*Gtw*). *G* = 0, if *Gtw* = 0.
- *GP* The average of *G* over a fixed number of instances.
- *Time*: average estimated running time in seconds. The symbol “+” is shown when the average running time is less than 0.001 s.
- *Pge*: percentage of transactions where *Gtw* = *Gtw*_*b*_ among all 1350 instances.

Table 3, represents the overview of results for all proposed algorithms. From this table, we can see that the best algorithm is *BIM* in 93.9% of cases, with an average gap of 0.03 and an average estimated running time of 0.003 s. The second best algorithm is *IML* with a percentage of 89.5%, an average gap of 0.05, and an average running time of 0.002 s.

**Table 3:** Overview of results for all proposed algorithms.

	*LTT*	*STT*	*IML*	*IMS*	*BIL*	*BIS*	*MIL*	*MIS*	*BIM*
*Pge*	32.4%	0.1%	89.5%	23.9%	81.0%	24.8%	60.3%	26.2%	93.9%
*GP*	0.47	0.88	0.05	0.56	0.10	0.54	0.24	0.53	0.03
*Time*	+	+	0.002	0.002	0.002	0.001	0.001	0.001	0.003

Table 4 represents the *GP* variation for all proposed algorithms. This latter table shows that for the best algorithm, *BIM* the best average gap of less than 0.001 is obtained when *n*_*T*_ = {15, 30}. However, the highest average gap of 0.09 is obtained when *n*_*T*_ = 40. For the *MIS* algorithm, the best *GP* value of 0.38 is obtained when *n*_*T*_ = 40. On the other hand, for the *IML* algorithm the best *GP* of 0.01 is reached when *n*_*T*_ = 15.

**Table 4:** Average gap *GP* variation for all proposed algorithms classified by *n*_*T*_.

*n*_*T*_	*LTT*	*STT*	*IML*	*IMS*	*BIL*	*BIS*	*MIL*	*MIS*	*BIM*
10	0.31	0.76	0.04	0.44	0.30	0.38	0.47	0.43	0.03
15	0.54	0.87	0.01	0.62	0.14	0.60	0.32	0.63	0.00
20	0.46	0.89	0.03	0.58	0.08	0.55	0.26	0.50	0.02
25	0.62	0.89	0.09	0.45	0.12	0.44	0.34	0.55	0.07
30	0.37	0.91	0.05	0.59	0.03	0.57	0.18	0.59	0.00
35	0.67	0.93	0.05	0.72	0.03	0.73	0.18	0.63	0.01
40	0.41	0.91	0.10	0.59	0.15	0.58	0.17	0.38	0.09
45	0.47	0.89	0.06	0.53	0.04	0.52	0.13	0.56	0.04
50	0.37	0.86	0.04	0.55	0.04	0.54	0.08	0.45	0.01

Table 5 shows the *GP* variation for all proposed algorithms classified by *n*_*b*_. For the best algorithm *BIM*, the minimum *GP* of less than 0.001 is obtained when *n*_*b*_ = 2 and the maximum *GP* of 0.06 is obtained when *n*_*b*_ = 3. For the *IML* algorithm the maximum *GP* is 0.09. It is clear that the maximum values of *GP* are obtained by *STT*.

**Table 5:** Average gap *GP* variation for all proposed algorithms classified by *n*_*b*_.

*n*_*b*_	*LTT*	*STT*	*IML*	*IMS*	*BIL*	*BIS*	*MIL*	*MIS*	*BIM*
2	0.51	0.93	0.01	0.12	0.01	0.16	0.17	0.35	0.00
3	0.63	0.92	0.09	0.65	0.13	0.64	0.29	0.57	0.06
4	0.55	0.86	0.07	0.65	0.11	0.62	0.31	0.52	0.04
5	0.27	0.89	0.04	0.78	0.13	0.74	0.20	0.73	0.01
6	0.38	0.78	0.05	0.62	0.14	0.56	0.22	0.46	0.03

**Fig 4:**
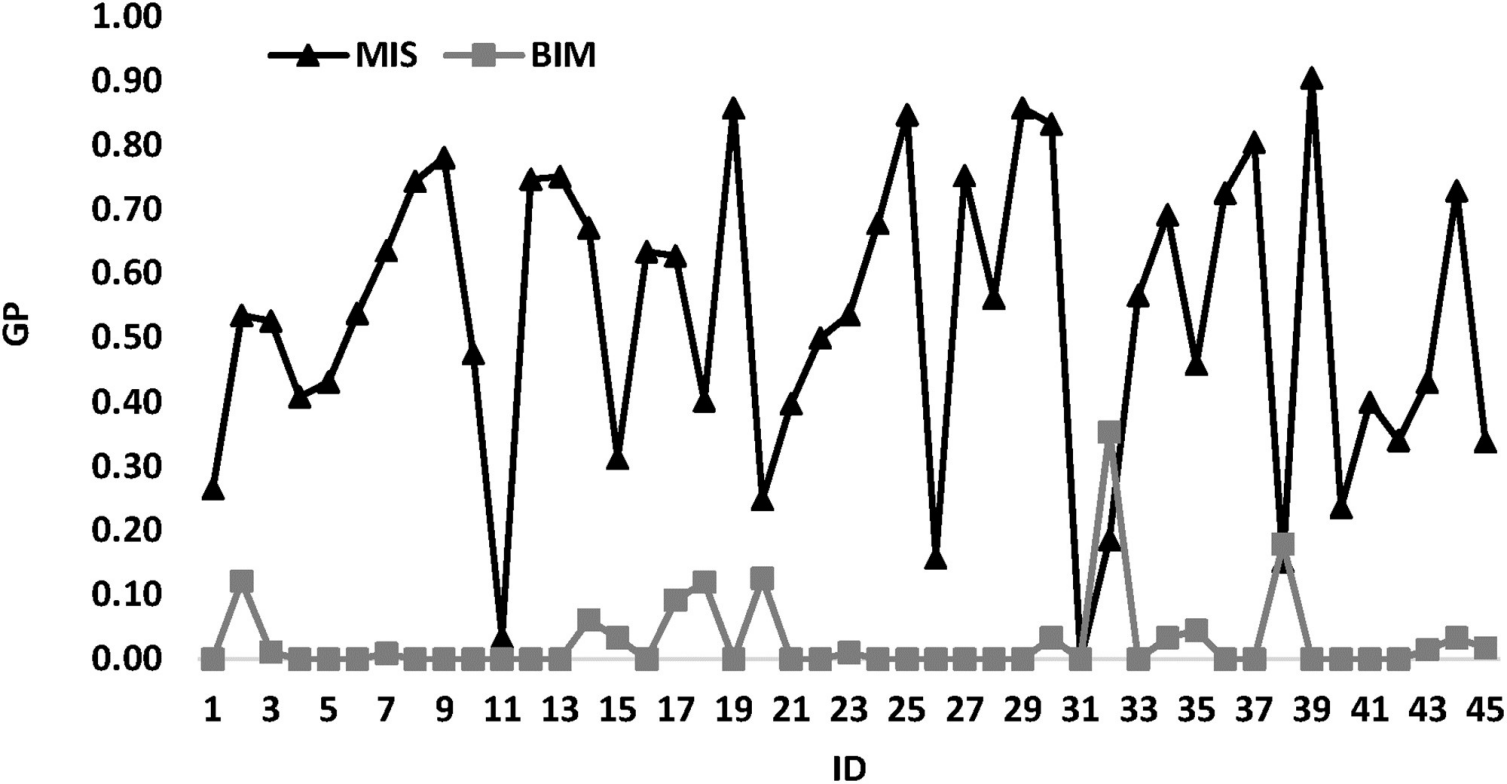
*MIS* and *BIM* comparison.

The pair (*n*_*T*_, *n*_*b*_) will be denoted by *ID*. So, for each (*n*_*T*_, *n*_*b*_) value, we have a new value of *ID*. The first *ID* value is 1. Fig 4 illustrates *MIS* and *BIM* comparison. This figure shows that almost the *MIS* curve is above the *BIM* curve. This means and confirms that *BIM* is better than *MIS*.

**Fig 5:**
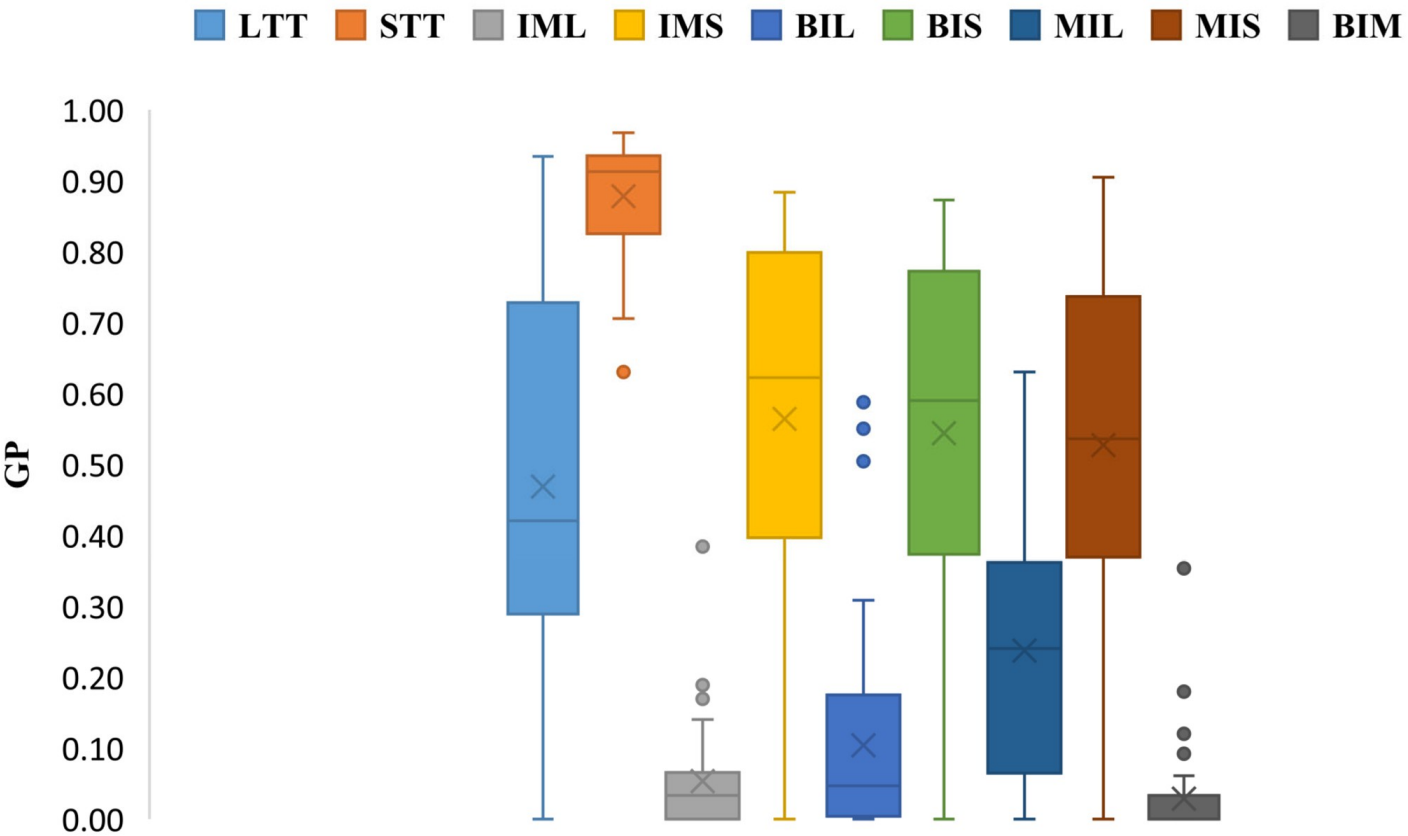
Comparison of average gap values for all algorithms based on *ID*.

Fig 5 shows the comparison of average gap values for all algorithms based on *ID*. This figure shows the performance of the algorithm *BIM*. This algorithm outperforms all other ones.

**Fig 6:**
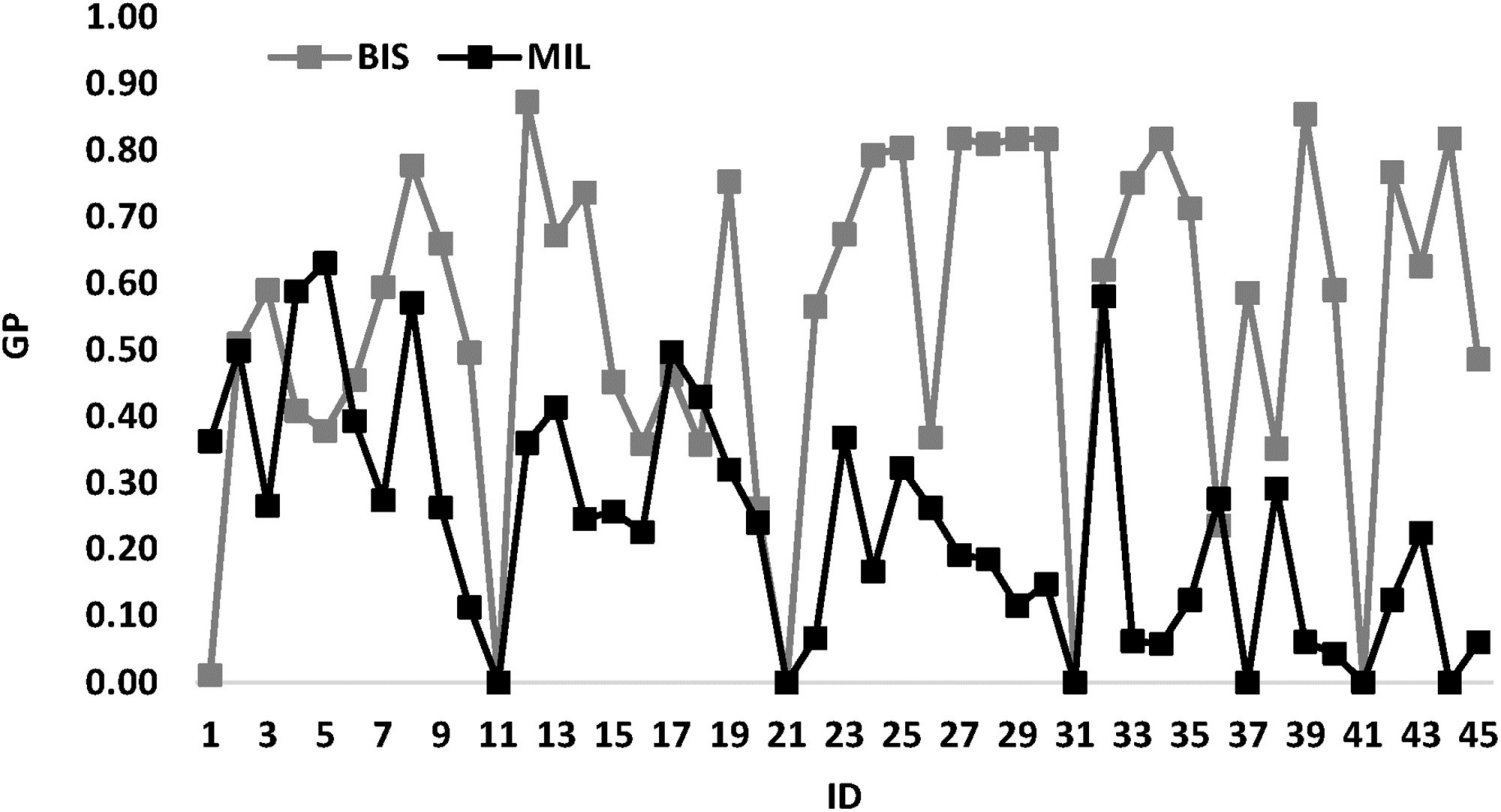
*BIS* and *MIL* comparison.

Fig 6 illustrates *BIS* and *MIL* comparison. This figure shows that almost the *MIL* curve is above the *BIS* curve. This means and confirms that *BIS* is better than *MIL*.

**Fig 7:**
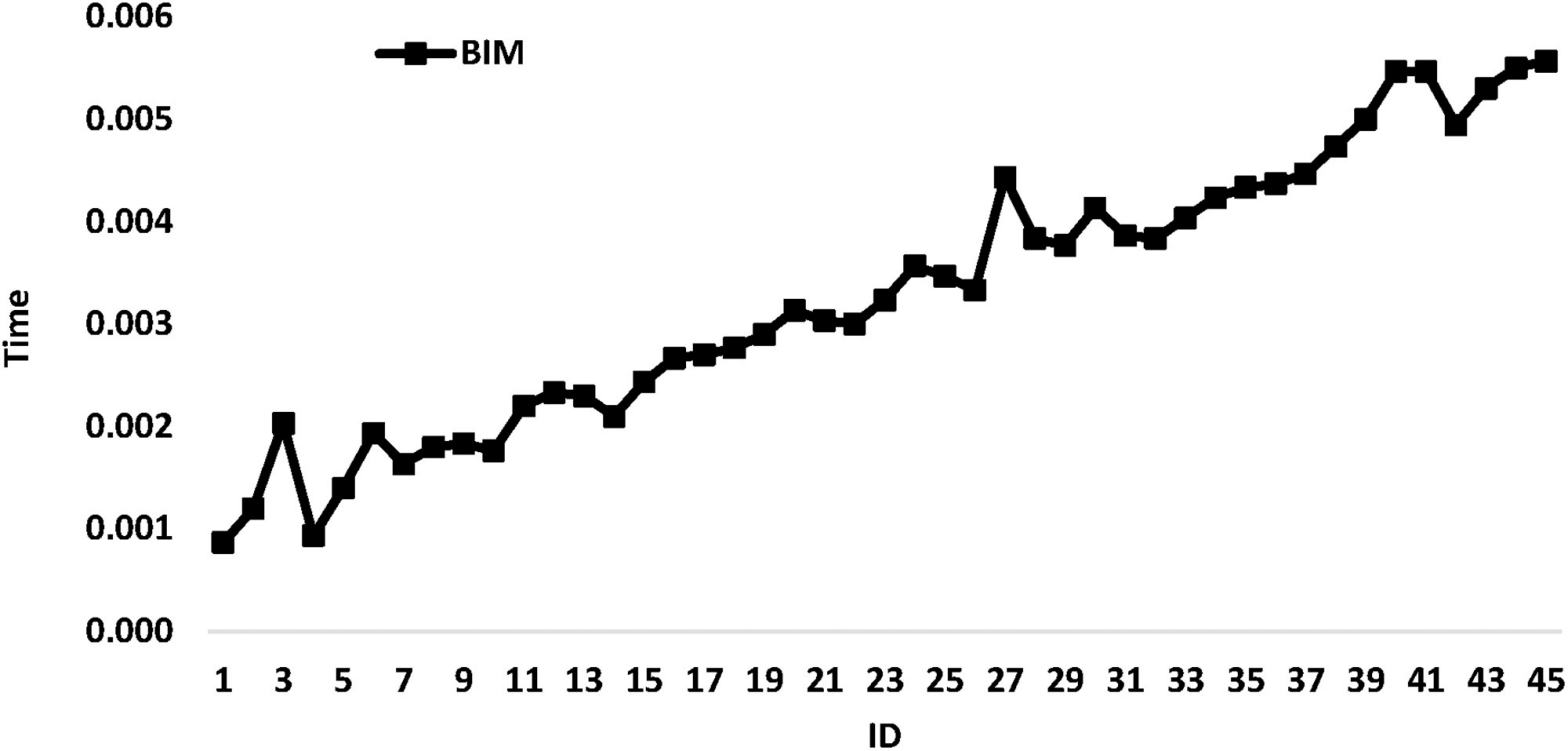
*BIM* running time variation when *ID* changes.

Fig 7 illustrates *BIM* running time variation when *ID* changes. Fig 7 shows that the running time increase when the *ID* increases.

For more details, Tables 6 and 7 show the average running time variation for all proposed algorithms classified by *n*_*b*_ and *n*_*T*_, respectively. Table 6 shows that the maximum running time of 0.005 s is reached for the *BIM* algorithm when *n*_*T*_ = 50. For *LTT* and *STT*, and for all values of *n*_*T*_, always the running time is less than 0.001 s. Table 7 shows that the maximum running time of 0.004 s is reached for the *BIM* algorithm when *n*_*b*_ = 6. For *LTT* and *STT* and for all values of *n*_*b*_, always the running time is less than 0.001 s.

**Table 6:** Average of running time variation for all proposed algorithms classified by *n*_*T*_.

*n*_*T*_	*LTT*	*STT*	*IML*	*IMS*	*BIL*	*BIS*	*MIL*	*MIS*	*BIM*
10	+	+	0.001	0.001	+	0.001	+	+	0.001
15	+	+	0.001	0.001	0.001	0.001	0.001	0.001	0.002
20	+	+	0.001	0.001	0.001	0.001	0.001	0.001	0.002
25	+	+	0.001	0.001	0.001	0.001	0.001	0.001	0.003
30	+	+	0.002	0.002	0.002	0.001	0.001	0.001	0.003
35	+	+	0.002	0.002	0.002	0.002	0.001	0.001	0.004
40	+	+	0.002	0.002	0.002	0.002	0.001	0.001	0.004
45	+	+	0.002	0.002	0.002	0.002	0.001	0.001	0.005
50	+	+	0.003	0.003	0.003	0.002	0.002	0.001	0.005

**Table 7:** Average of running time variation for all proposed algorithms classified by *n*_*b*_.

*n*_*b*_	*LTT*	*STT*	*IML*	*IMS*	*BIL*	*BIS*	*MIL*	*MIS*	*BIM*
2	+	+	0.002	0.002	0.002	0.001	0.001	0.001	0.003
3	+	+	0.002	0.002	0.001	0.001	0.001	0.001	0.003
4	+	+	0.002	0.002	0.002	0.002	0.001	0.001	0.003
5	+	+	0.002	0.002	0.002	0.001	0.001	0.001	0.003
6	+	+	0.002	0.002	0.002	0.001	0.001	0.001	0.004

Table 8 shows the contrast estimation results for all proposed algorithms. The importance of this test is based on the estimation of the contrast between medians of instances of results considering all pairwise comparisons. The test obtains a quantitative difference between gaps computed through medians between two algorithms over all instances [58]. Table 8 shows the estimations computed for each proposed algorithm. Focusing the attention on the rows of Table 8, we can see the performance of *BIM* (all its related estimators are negative).

**Table 8:** Contrast estimation results for all proposed algorithms.

	*LTT*	*STT*	*IML*	*IMS*	*BIL*	*BIS*	*MIL*	*MIS*	*BIM*
*LTT*	0	-0.268	0.067	-0.086	-0.016	0.017	-0.227	0.134	-0.369
*STT*	0.268	0	0.335	0.182	0.252	0.285	0.042	0.402	-0.100
*IML*	-0.067	-0.335	0	-0.153	-0.083	-0.050	-0.293	0.067	-0.435
*IMS*	0.086	-0.182	0.153	0	0.070	0.103	-0.141	0.220	-0.283
*BIL*	0.016	-0.252	0.083	-0.070	0	0.033	-0.210	0.150	-0.352
*BIS*	-0.017	-0.285	0.050	-0.103	-0.033	0	-0.243	0.117	-0.385
*MIL*	0.227	-0.042	0.293	0.141	0.210	0.243	0	0.360	-0.142
*MIS*	-0.134	-0.402	-0.067	-0.220	-0.150	-0.117	-0.360	0	-0.502
*BIM*	0.369	0.100	0.435	0.283	0.352	0.385	0.142	0.502	0

The proposed algorithms show their performance in running time. Indeed, the most higher running time is 0.005 s. This means the polynomial time of the proposed algorithms. The experimental results show the non-dominance of the proposed algorithms. This can allow researchers to make a combination of the proposed algorithms to propose new ones. The proposed algorithms can be utilized in a branch and bound algorithm to develop an exact solution to the studied problem. This is because the time execution of the best algorithm is very remarkable and efficient. So, the burning of a node in the tree doesn’t take time and memory to expand the other possibilities. Other methodologies that can be used to achieve the objective in relation to this work are:

- Applying some met-heuristics and considering the proposed algorithms as heuristics and initial solutions.
- Search the square of difference gap instead of the proposed objective given in Eq 1.
- Other new metrics can be considered like quadratic difference (*RMSE*) between the *AGP*s for all algorithms.

The four best proposed-algorithms are *IML*, *BIL*, *MIL*, and *BIM*. The results illustrated in tables and figures show the performance of the algorithms. The application of randomization has a remarkable impact on the performance of the algorithm. Indeed, these four algorithms are based on the randomization method. This means that the randomization method proves its efficacy in the proposed problem.

## 7 Conclusion

The application of technology in different domains is a crucial point. However, optimizing the utilization of technological information is very important to save time and money. This paper dealt with the solution to the problem related to the transactions that can be treated in the blocks of the blockchain. This problem is an NP-hard one. This problem can be solved by proposing an approximate solution. It is important to give an approximate solution to the studied problem in polynomial time. Nine algorithms are proposed to solve the studied problem in polynomial time. These algorithms are based on the dispatching-rules method, randomization approach, clustering algorithms, and iterative method. The results show that the best algorithm proposed in this paper is best-mi-transactions iterative multi-choice with 93.9% in an average running time of 0.003 s. The proposed algorithms give several approximate solutions in a remarkable running time. The limitation of this work is the comparison of the best-proposed algorithm to a valid lower bound of the studied problem. Four future axes can be extended for this research. The first axe is to develop an optimal solution to the studied problem. The proposed algorithms can be utilized to obtain the exact solution in a decision tree. The second future axe is to test the proposed algorithms over extended classes of instances and determine some hard classes of instances. The third future axe is to use the proposed algorithms in several meta-heuristics to enhance the solutions. The last future axe is the incorporation of the proposed algorithm in the future to solve other kinds of problems in cloud management or network routing.
